# Plasma proteomics and carotid intima-media thickness in the UK biobank cohort

**DOI:** 10.3389/fcvm.2024.1478600

**Published:** 2024-10-02

**Authors:** Ming-Li Chen, Pik Fang Kho, Rodrigo Guarischi-Sousa, Jiayan Zhou, Daniel J. Panyard, Zahra Azizi, Trisha Gupte, Kathleen Watson, Fahim Abbasi, Themistocles L. Assimes

**Affiliations:** ^1^ Department of Medicine, Division of Cardiovascular Medicine, Stanford University School of Medicine, Stanford, CA, United States; ^2^ Palo Alto Veterans Institute for Research (PAVIR), Stanford, CA, United States; ^3^ Department of Genetics, Stanford University School of Medicine, Stanford, CA, United States; ^4^ Department of Psychiatry and Behavioral Sciences, Stanford University School of Medicine, Stanford, CA, United States; ^5^ Stanford Cardiovascular Institute, Stanford University School of Medicine, Stanford, CA, United States

**Keywords:** plasma proteomics, carotid intima-media thickness, UK biobank, atherosclerosis, risk factors

## Abstract

**Background and aims:**

Ultrasound derived carotid intima-media thickness (cIMT) is valuable for cardiovascular risk stratification. We assessed the relative importance of traditional atherosclerosis risk factors and plasma proteins in predicting cIMT measured nearly a decade later.

**Method:**

We examined 6,136 UK Biobank participants with 1,461 proteins profiled using the proximity extension assay applied to their baseline blood draw who subsequently underwent a cIMT measurement. We implemented linear regression, stepwise Akaike Information Criterion-based, and the least absolute shrinkage and selection operator (LASSO) models to identify potential proteomic as well as non-proteomic predictors. We evaluated our model performance using the proportion variance explained (*R*^2^).

**Result:**

The mean time from baseline assessment to cIMT measurement was 9.2 years. Age, blood pressure, and anthropometric related variables were the strongest predictors of cIMT with fat-free mass index of the truncal region being the strongest predictor among adiposity measurements. A LASSO model incorporating variables including age, assessment center, genetic risk factors, smoking, blood pressure, trunk fat-free mass index, apolipoprotein B, and Townsend deprivation index combined with 97 proteins achieved the highest *R*^2^ (0.308, 95% C.I. 0.274, 0.341). In contrast, models built with proteins alone or non-proteomic variables alone explained a notably lower *R*^2^ (0.261, 0.228–0.294 and 0.260, 0.226–0.293, respectively). Chromogranin b (CHGB), Cystatin-M/E (CST6), leptin (LEP), and prolargin (PRELP) were the proteins consistently selected across all models.

**Conclusion:**

Plasma proteins add to the clinical and genetic risk factors in predicting a cIMT measurement. Our findings implicate blood pressure and extracellular matrix-related proteins in cIMT pathophysiology.

## Introduction

Ultrasound derived carotid intima media thickness (cIMT) is well-established tool used to noninvasively measure the thickness of the carotid artery in high-resolution (1). Suggested applications of cIMT include using the measurement to improve cardiovascular risk stratification and the prediction of future cardiovascular events over that possible with traditional risk factors alone, as well as the assessment of the efficacy of cardiovascular drugs (2–5). Nevertheless, the reproducibility of cIMT images has long been its major limitation because performing cIMT measurements and image analysis requires standardized equipment and protocol, as well as a trained sonographer (6).

High throughput profiling of circulating proteins in the plasma has emerged as a useful approach to improved risk prediction as well as to provide novel insights on the biology of complex human traits (7). When integrated with genetic and environmental determinants of diseases, proteomic-based models can outperform clinical risk factor models in predicting cardiovascular events (8, 9). However, it is unknown to what extent plasma proteomics correlate with cIMT, and whether they improve its prediction over clinical measures.

We aimed to evaluate the potential of a signature of plasma proteins to predict the measurement of cIMT nearly a decade later in a generally healthy population. To accomplish this task, we leveraged data from the UK Biobank (UKBB) to investigate individuals with measurements of circulating proteins in the plasma, genetic variation, lifestyle factors, and health outcomes, and ultrasound imaging of the carotids (10).

## Methods

### Study population

**Figure 1:**
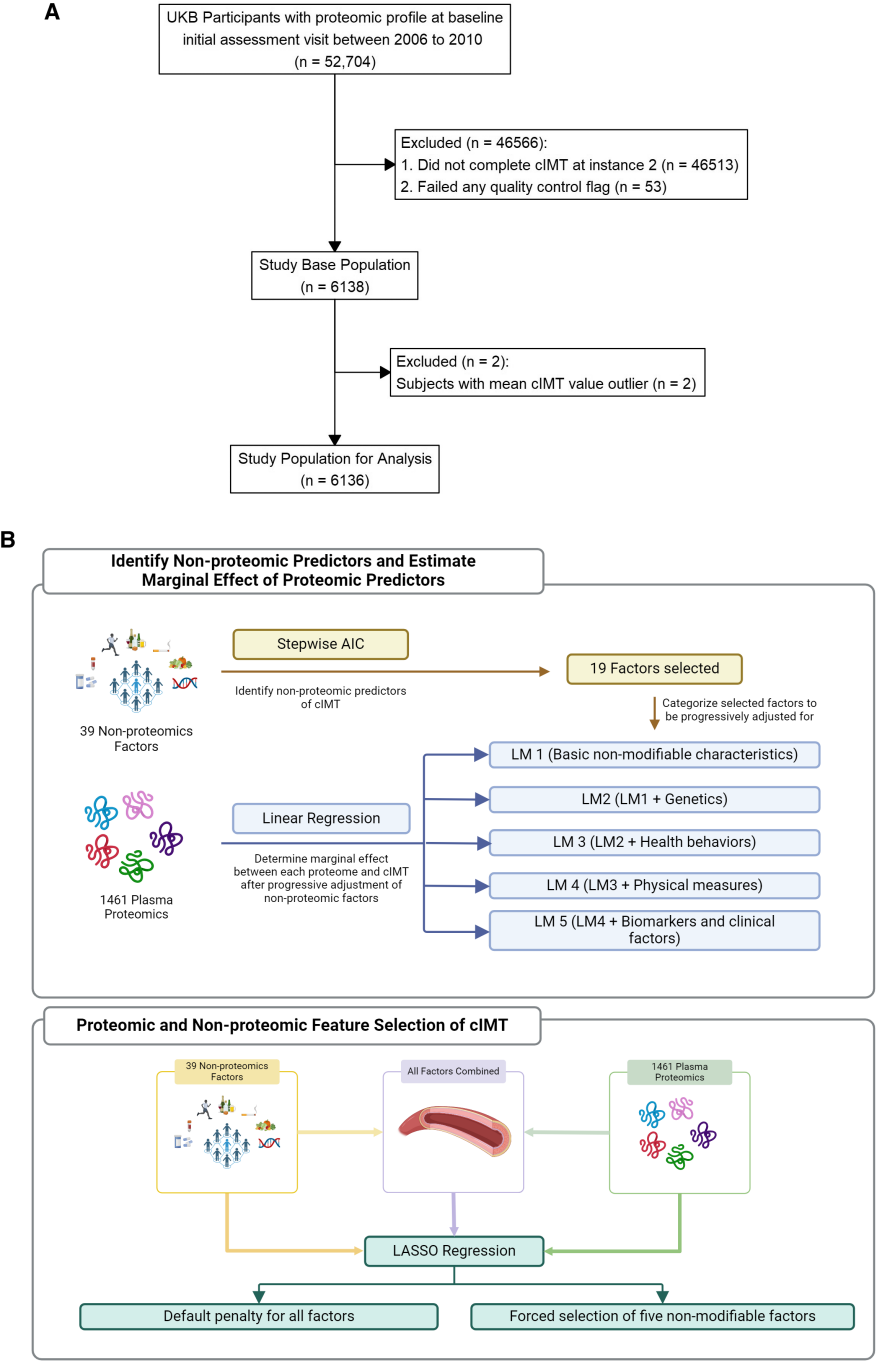
**(A)** Study population. **(B)** Analysis workflow.

We analyzed a sub cohort of UKBB participants of the UKBB who underwent both high-throughput proteomic profiling as well as a cIMT study (Figure 1A). The study design, as well as the methods of data collection of the UKBB is described elsewhere in detail (11, 12). Briefly, participants’ baseline visit occurred between 2006 and 2010 (Instance 0) during which baseline characteristics and a personal health history were collected using touchscreen questionnaires, physical measures, and verbal interviews. Blood was also drawn and banked. A subset of participants returned for an imaging visit starting in 2014 (Instance 2). The UKBB received ethical approval from the National Information Governance Board for Health and Social Care and the Northwest Multicenter Research Ethics Committee. All participants provided their written informed consent at baseline.

### Plasma proteomic biomarkers

A total of 1,463 circulating proteins in plasma were previously measured using the antibody-based Proximity Extension Assay by Olink. Extensive quality control of protein measures was implemented by the UKBB using a set of predefined criteria as previously described (10). Levels were normalized using a two-step approach of within-batch and across-batches intensity normalization (13). We excluded data for two proteins with a high missing rate across our study samples (NPML with 74% and PCOLCE with 63% missing). The values were log-transformed and standardized before analysis. Missing normalized protein expression (NPX) values were imputed with their mean values.

### Outcome

The cIMT was measured at the imaging visit (instance 2) in four angles using the CardioHealth Station ultrasound system and UKBB predefined standards (14, 15). We included only individuals with cIMT measurements that did not fail any quality control indicators in our analyses. The average value of cIMT of the four angles was taken as the primary outcome measure in this study. The distribution of cIMT measures was acceptable (skewness = 0.89) after removing outliers of larger than five standard deviations (SD) from the mean. A detailed description of how the cIMT measurement was acquired is included in the Supplementary File.

### Selection of non-proteomic covariates

Potential non-proteomic covariates were selected based on a careful literature review focused on the identification of previously established and/or suspected non-proteomic predictors of cIMT. We considered the following groups of variables documented at baseline: demographic characteristics (sex, age when attending the baseline assessment and the imaging assessment, self-reported race/ethnicity, Townsend deprivation index, and assessment center location), anthropometric characteristics (waist circumference, body mass index, calculated whole body and trunk fat as well as fat-free mass index estimated through whole-body bio-impedance), health behaviors (alcohol consumption, ever smoked, and categorized physical activity groups), medical conditions, medications, physical measures (blood), nine biochemistry markers, five genetic principle components, and four polygenic risk scores (PRS) of health traits related to cardiometabolic risk from the “Standard PRS Set” UKBB resource (16, 17).

### Statistical analyses

We first determined the marginal association between each non-proteomic factor and cIMT. Subsequently, we selected five non-modifiable characteristics (sex, age including at baseline and at the imaging visit, center of imaging visit, and self-reported ethnicity) to define a minimally adjusted model. We then applied an Akaike information criterion (AIC) based stepwise model selection algorithm to the remaining non-proteomic covariates of interest to identify a subset that improved the AIC for cIMT in a multivariable variable framework. Non-proteomic variables that were selected were then further grouped into four to allow for multivariate testing of five linear regression models (LM) with increasingly progressive covariate adjustment: (1) the five basic non-modifiable characteristics (LM 1), (2) model 1 plus multiple PRS (LM 2), (3) model 2 plus health behaviors (LM 3), (4) model 3 plus physical measures (LM 4), and (5) model 4 plus medical conditions (LM 5). More detailed analysis plan is described in Supplementary File.

Next, we used the least absolute shrinkage and selection operator (LASSO) regression to identify a subset of plasma proteomic measures predictive of cIMT alone, or in combination with non-proteomic covariates. We built the LASSO model using 10-fold cross-validation after creating a randomly selected training (70%) and testing (30%) subset. Factors were centered and scaled before analysis. We trained six LASSO models, with a pair of models drawing only from protein levels, a pair drawing only from non-proteomic variables, and a pair drawing from a combination of both. Within each pair, one model was trained after forcing the five non-modifiable characteristics of model 1 without penalty. Lastly, we quantified model improvement after adding proteomic measures to other non-proteomic variables by evaluating improvements in variance explained (*R*^2^) and reductions in the root mean square error (RMSE) in the testing set. The 95% confidence intervals were generated by bootstrapping 1,000 samples. The Benjamini-Hochberg False Discovery Rate (FDR) threshold of 0.05 was used to account for multiple testing of proteomic-cIMT associations.

Sensitivity analyses were performed excluding protein measurements with a high proportion of NPX values flagged as being below the limit of detection (LOD) corresponding to each sample's plate ID. We repeated the six LASSO models in the same training and testing population after excluding proteins with below LOD measures for >25%, >10%, and >3% of the participants, respectively. 95% confidence intervals were generated by bootstrapping 1,000 samples. Statistical analyses were performed with R, version 4.1.1.

### Gene set enrichment analysis

We implemented gene set enrichment analysis (GSEA) to investigate the possible enrichment of biochemical pathways from proteins identified in the previous screening method (18–22). For this analysis, the normalized enrichment score (NES) was the primary statistic. We considered two levels of significance, an FDR q value of less than 0.05 and a more liberal FDR q value of less than 0.25 if no gene sets met the more stringent threshold of significance. GSEA software (v4.3.2) was used for this analysis.

## Results

### Cohort characteristics

A total of 1,461 plasma proteins levels in 6,136 participants were included in our analysis (Figure 1A). The baseline characteristics of the study population are shown in Table 1. The mean time from baseline visit to imaging visit for cIMT visit was approximately 9 years with a little less than half of the participants being men. Summary statistics of the full list of covariates considered are included in Supplementary Table 1 while additional annotation of proteins measured including their summary statistics within the study cohort is included in Supplementary Table 2.

**Table 1:** Demographic and clinical characteristics of total study population.

	Overall
***n***	6,136
Male (%)	2,901 (47.3)
Age at baseline	54.39 (7.86)
Age at imaging visit	63.61 (8.08)
Ethnicity group (%)	
Asian	67 (1.1)
Black	51 (0.8)
Mixed	41 (0.7)
Other	52 (0.8)
White	5,925 (96.6)
Townsend deprivation index at recruitment [median (IQR)]	−2.56 [−3.88, −0.24]
Ever smoked (%)	3,597 (58.6)
Alcohol intake frequency (%)	
Never or missing	296 (4.8)
One to three times a month or special occasions only	1,154 (18.8)
Once or twice a week	1,564 (25.5)
Three or four times a week	1,727 (28.1)
Daily or almost daily	1,395 (22.7)
Physical activity category (%)	
Missing	884 (14.4)
Low	981 (16.0)
Moderate	3,648 (59.5)
High	1,507 (24.6)
Waist circumference (cm)	87.61 (12.43)
BMI (kg/m^2^)	26.48 (4.21)
Whole body fat mass index (kg/m^2^)	8.10 (3.19)
Whole body fat-free mass index (kg/m^2^)	18.40 (2.51)
Trunk fat mass index (kg/m^2^)	4.47 (1.64)
Trunk fat-free mass index (kg/m^2^)	10.25 (1.26)
SBP (mmHg)	135.96 (18.54)
DBP (mmHg)	80.99 (10.55)
LDL direct [mmol/L, median (IQR)]	3.50 [2.96, 4.09]
HDL cholesterol [mmol/L, median (IQR)]	1.43 [1.21, 1.71]
TG [mmol/L, median (IQR)]	1.38 [0.98, 1.98]
Cholesterol [mmol/L, median (IQR)]	5.63 [4.94, 6.39]
HbA1c [mmol/mol, median (IQR)]	34.50 [32.10, 37.00]
Apolipoprotein A [g/L, median (IQR)]	1.52 [1.36, 1.70]
Apolipoprotein B (g/L, median [IQR]	1.00 [0.86, 1.17]
Lipoprotein A [nmol/L, median (IQR)]	19.96 [9.34, 57.83]
C-reactive protein [mg/L, median (IQR)]	1.05 [0.54, 2.10]
Vascular/heart problems diagnosed by doctor (%)	1,278 (20.8)
Diabetes diagnosed by doctor (%)	137 (2.2)
Blood pressure medication (%)	497 (8.1)
Cholesterol lowering medication (%)	492 (8.0)
Statin use (%)	628 (10.2)
Aspirin use (%)	661 (10.8)
PRS for CVD	−0.15 (0.95)
PRS for HTN	−0.11 (0.93)
PRS for ISS	−0.08 (0.91)
PRS for T2D	−0.22 (0.95)
Follow up time (days)	3,369.91 (697.15)
Mean cIMT (micrometers)	680.67 (123.99)

All continuous measurements were documented in mean (SD) unless otherwise specified. IQR, interquartile range; BMI, body mass index; SBP, systolic blood pressure; DBP, diastolic blood pressure; TG, triglycerides; HbA1c, glycated hemoglobin; PRS, polygenic risk score; CVD, cardiovascular disease; HTN, hypertension; ISS, ischemic stroke; T2D, type 2 diabetes; PC, principal components.

### Covariate selection with AIC and standard linear regression of proteins

From a total of 39 non-proteomic factors analyzed, the most predictive covariates in univariate regression models were those related to age (either at baseline or at the time of imaging visit) with each explaining nearly one fifth of the variance (*R*^2^) of cIMT (Supplementary Table 3). In contrast, the next strongest non-modifiable predictor was sex which explained only between 2% and 3% of *R*^2^. The most predictive modifiable covariate was systolic blood pressure explaining about 6%–7% of *R*^2^. A total of 19 out of 39 potential covariates were selected using the AIC based stepwise model selection algorithm and grouped into the five LM (Supplementary Table 4). To benchmark the predictive ability of these non-proteomic variables selected in the previous five multiple LMs, we calculated the variance explained (*R*^2^) of each model in the testing dataset. As more explanatory demographic, genetic, and phenotypic variables were included, especially blood pressure and anthropometric measures, the *R*^2^ increased from 0.211 (95%C.I. 0.178, 0.244) in LM 1 to 0.256 (95%C.I. 0.223, 0.290) in the fully adjusted LM 5 (Supplementary Figure 1).

Next, we screened all proteins to identify the subset of proteins marginally associated with cIMT with progressive adjustment of our non-proteomic covariates identified. When LM 1 through 5 were applied in multivariable model with each protein, we identified 374, 289, 310, 11, and 4 proteins at FDR < 0.05. The 11 proteins in LM 4 that were significant included Secretogranin-1 (CHGB), Cystatin-M (CST6), formin-like protein 1 (FMNL1), interleukin-17D (IL17D), leptin (LEP), matrix extracellular phosphoglycoprotein (MEPE), phosphoprotein associated with glycosphingolipid-enriched microdomains 1 (PAG1), platelet-activating factor acetylhydrolase (PLA2G7), phospholipid transfer protein (PLTP), prolargin (PRELP), and signaling threshold-regulating transmembrane adapter 1 (SIT1) while the four proteins that remained significant in LM 5 where CHGB, CST6, LEP, and PRELP (Supplementary Table 5).

### LASSO regression models

**Figure 2:**
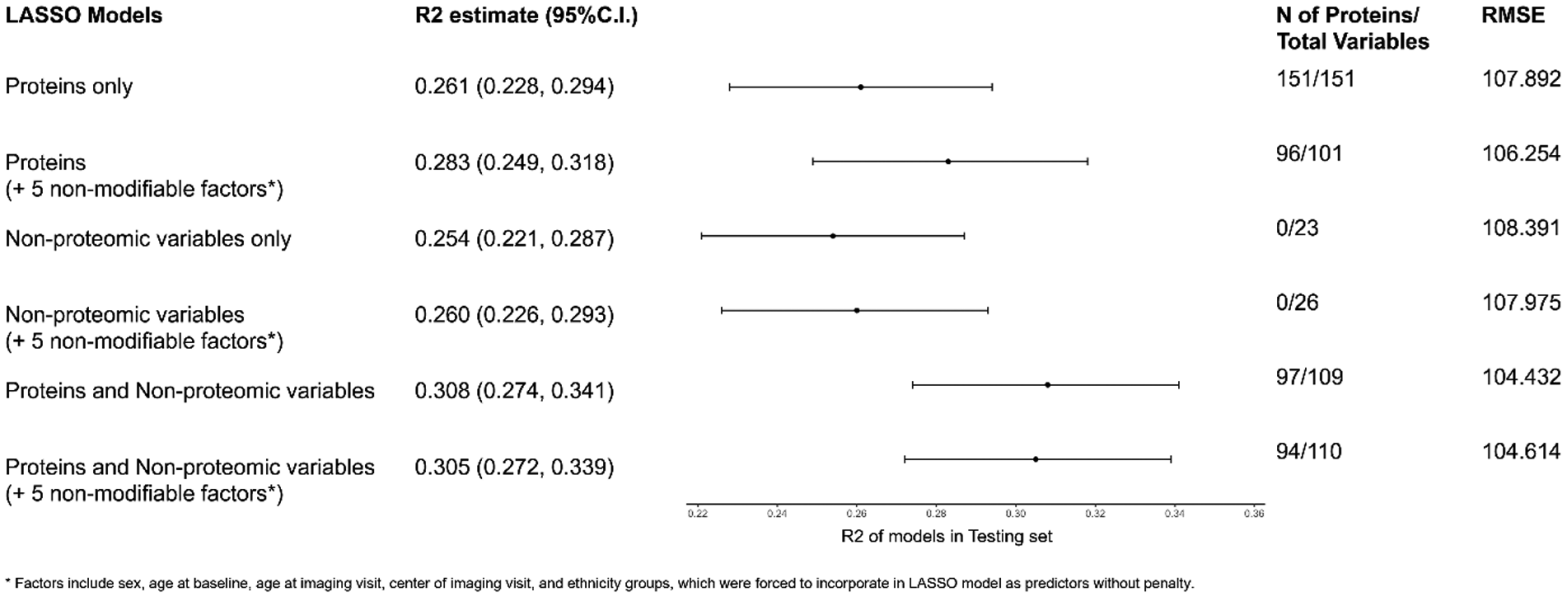
Variance explained (*R*^2^) of the LASSO regression models ^a^performed in the test dataset. ^a^Selected covariates include sex, age at baseline, age at imaging visit, center of imaging visit, and ethnicity groups. Models with selected covariates were forced to incorporate all selected covariates as predictors. Since a full rank parameterization was used in LASSO, different levels of categorical variables (except the reference group) counted separately toward the number of total variables. LASSO, least absolute shrinkage and selection operator; RMSE, root mean square error; min, minimum; N, number.

Using LASSO regression, we noted the following trends (Figure 2; Supplementary Table 6). In models that allowed for the selection of proteins, a range of 94–151 proteins were selected while in models with non-proteomic covariates, a range of 12–16 non-proteomic covariates were selected. A model restricted to non-proteomic measures explained the least variance (*R*^2^ = 0.254). A model including the five non-modifiable covariates in addition to proteins increased the variance explained (*R*^2^ = 0.283) to a degree that was comparable to models built from a combination of non-proteomic and proteomic predictors. A LASSO model combining 97 proteins with 12 non-proteomic factors performed the best in the testing set (*R*^2^ = 0.308). Lastly**,** we observed significant overlap of the 95% confidence intervals of all models that included at least some non-proteomic covariates. The cross-validation mean squared error plots of all six LASSO models for the study population are shown in Supplementary Figure 2. A summary of all proteomic and non-proteomic predictors selected by the linear regression or LASSO models is shown in Supplementary Tables 7, 8.

### Sensitivity analysis LASSO regression models

A total of 175 out of 1,461 proteins had more than 25% of their measures below LOD, while 244 proteins had more than 10% and 472 proteins had more than 3% below LOD. When excluding these proteins, the variance explained was largely unchanged compared to that observed in the main analyses (Supplementary Table 9).

### Gene set enrichment analysis

In GSEA, while no gene set was significant at FDR-q <0.05, we found 9 upregulated canonical pathways, 3 downregulated canonical pathways, and 3 Hallmark gene sets reached FDR <0.25 (Supplementary Table 10 and Supplementary Figures 3, 4).

## Discussion

We leveraged the large-scale profiling of nearly 1,500 proteins in the plasma of >6,000 participants of the UK Biobank study who also underwent ultrasound imaging of their carotids to provide an initial estimate of the relative importance of proteomic and non-proteomic measures in predicting a cIMT measurement and in understanding the pathophysiologic link behind an increased rate of thickening and the future risk of atherosclerosis related complications in both proximal and more distant arterial trees (23, 24).

With respect to prediction of cIMT, we document several interesting trends. First, as has been shown by others in the same cohort (25), age was by far the single strongest predictor of cIMT explaining three times more *R*^2^ than the next best non-proteomic predictor, systolic blood pressure, and about two thirds the maximal *R*^2^ explained by our best LASSO model. Second, approximately one quarter of all proteins measured were marginally associated with cIMT in the setting of our minimally adjusted model. Third, a model restricted to approximately 150 proteins performed as well as the best predictive model using clinical variables explaining a little over a quarter of the *R*^2^ of cIMT. Lastly, the addition of approximately 100 proteins through a LASSO model notably increased the absolute *R*^2^ explained from ∼25% to ∼30%, or a ∼20% relative increase. While the added predictive value of proteomic profiling was not dramatic, our findings open the door to the future development of a simple, blood-based, proteomic or multi-omics signature that may either enhance or serve as a good proxy to cardiovascular risk assessment with a cIMT measurement (26, 27).

We highlight several modifiable factors that were significant in our multivariate linear regression models and were also consistently selected by LASSO including blood pressure, smoking, apolipoprotein B, and trunk fat-free mass index. Despite the very high correlation of the same mass indices between different body parts, the fat-free mass indices were repeatedly selected over fat mass indices by several models. These findings confirm prior studies demonstrating the importance of traditional risk factors of atherosclerosis in determining cIMT (28–30), as well as more recent studies exploring the relative contributions of correlated measures of body composition (29, 31–33). A robust signature of modifiable risk factors within circulating plasma proteins is also evident in our progressive multivariate linear regression models given the number of marginally associated proteins dropped dramatically with the progressive addition of more non-proteomic measures.

Four proteins remained significantly associated with cIMT after a comprehensive adjustment with non-proteomic variables that included non-modifiable factors, PRS, health behaviors, physical measures, biomarkers and medical history. The first was CHGB or Chromogranin B (also known as Secretogranin-1) (34, 35). CHGB is a member of the granin family of neuroendocrine secretory proteins, commonly found in the secretory vesicles of neurons and endocrine cells and plays a significant role in the regulation of neuroendocrine secretions (34, 35). While a related protein, Chromogranin A, serves as a precursor to Catestatin, a modulator of the neuroendocrine system that has antihypertensive properties through the inhibition of the release of catecholamines, such a relationship does not exist between Chromogranin B and blood pressure (34, 35).

CST6 (also known as Cystatin M/E or Cystatin E/M), is a secreted protein that is part of the type 2 cystatin subfamily. Its primary role is to act as an inhibitor of lysosomal cysteine proteases including cathepsins and legumain by forming high-affinity reversible complexes (36, 37). CST6 is known to contribute to the homeostatis of the skin, but also been implicated in cancer biology, as it is often found to be downregulated, and sometimes completely silenced, in various cancer types such as breast cancer, melanoma, and lung cancer (36, 37). When CST6 isn't functioning properly, it can lead to changes in the proteolysis of tissue structures, which could potentially speed up the spread of cancer cells. These extracellular effects on the tumor microenvironment may also be reflected some effects on the extracellular matrix within the carotid media. Of note, a prior study found Cystatin N/E protein expressions to be positively correlated with symptoms among patients with carotid artery disease (38).

The third protein, the adipocyte derived hormone leptin (LEP), has more obvious connections to cIMT through established effects on blood pressure (39). These effects are facilitated by the enhancement of the sympathetic nervous system, initiated by the stimulation of pro-opiomelanocortin (POMC) neurons in the hypothalamic arcuate nucleus. These neurons project to the periventricular nucleus, leading to the release of the *α*-melanocortin stimulating hormone, which subsequently activates melanocortin receptors on presympathetic neurons. Separately, leptin causes sodium retention through a direct action on the renal tubules as well as the renin–angiotensin–aldosterone system via stimulation of aldosterone release in the adrenal cortex (39).

The final protein, PRELP, is a secreted protein found in the aortic extracellular environment (ECM)associated with pre-atherosclerotic lesions (40, 41). PRELP also binds the basement membrane protein heparan sulfate proteoglycan perlecan (HSPG2), which regulates lipid deposition (42). In our analysis, HSPG2 was also shown to be positively associated with cIMT. Lastly, the highest bulk tissue gene expression for PRELP is found in vascular tissues (aorta, coronary artery, and tibial artery) and principle single cell sources of this protein include the fibroblast, endothelial cells, pericytes, and smooth muscle cells (43, 44). Together, these findings suggest possible contributory role of PRELP in maintaining the structural integrity and remodeling of the ECM within the cardiovascular system including the carotid-intima media region.

We compared our findings to a recently published association analysis between cIMT measurements and the levels of 1,095 proteins measured in blood using the SOMAscan Platform in a subset of 893 participants of the KORA F4 population-based cohort study (45). The researchers found four proteins to be associated with cIMT in a basic age-sex adjusted model, cytoplasmic protein NCK1 (NCK1), insulin-like growth factor-binding protein 2 (IGFBP2), growth hormone receptor (GHR), and GDNF family receptor alpha-1 (GFRA1), with only NCK1 remaining significant in their fully adjusted model (45). In our study, neither NCK1 and GHR were measured using Olink, but both GFRA1 and IGFB2 were significant in our models 1, 2, and 3. A look-up of our top findings in KORA F4 revealed results for only two out of our four top proteins, LEP and CST6 with LEP showing nominally significance in both models (45). This degree of replication between studies is not unexpected given differences in design (cross-sectional vs. longitudinal), power (893 vs. 6,183 subjects), analytic approach to declaring significance (Bonferroni vs. FDR) and measurement platform used (SOMA scan vs. OLINK). We note that multiple studies have shown a bimodal distribution of correlations between the two platforms for proteins measured in both platforms (46–49). While the correlations for LEP have been on the high end, the correlations for CST6 have been on the low end.

The main strengths of our study are the standardized protocols for cIMT, the large sample size, and the comprehensive proteomic profiling using a high-throughput platform known to maintain a high specificity. Limitations are several as well. First, the long time between the time of the proteomic and the cIMT measurements may have limited our ability to correlate more strongly with cIMT. Nevertheless, the significant proteins identified in our study may serve as candidate biomarkers for early prediction of cIMT, conditional on age and other cardiovascular risk factors. Secondly, since no atherosclerotic plaque was measured in the UKBB, the association between proteins and plaque will require additional studies to confirm or refute their linkage to atherosclerosis related health traits. Thirdly, the healthy volunteer bias and the predominantly white population (12) may limit the generalizability of our findings and mandate future studies that are both more ancestrally and socioeconomically diverse. Lastly, we acknowledge two significant challenges to conducting our GSEA. First, many gene sets were only partially represented by protein measures in plasma given only ∼1,500 protein measures were available. Second, additional substantial additional statistical noise is likely introduced by moving out of cell and representing genes through their plasma levels of proteins derived from potentially all tissues in the body rather than their expression levels intracellularly or by genetic variation affecting their expression levels. Thus, we suspect our analyses are substantially underpowered. Nevertheless, we provide some insights on potentially relevant biology represented by our highest ranked genes sets in the supplementary appendix.

In summary, we found proteomic profiles in plasma achieve notable incremental prediction over non-proteomic factors of cIMT. This incremental prediction is achieved despite the separation of the two sets of measures (plasma proteins and cIMT) by nearly a decade. Our findings suggest proteomic signatures, possibly combined with other -omic measurements in blood, may one day enhance or serve as a reliable cross-sectional surrogate of a direct measurement of cIMT. Among our most robustly associated proteins, we note proteins involved in the modification of core mechanisms determining cIMT such as blood pressure, but also evidence of the role of multiple cell types active in the vascular wall. Future studies are needed to provide a clearer understanding of these associations, and the relevant mechanisms involved.

## Data Availability

Publicly available datasets were analyzed in this study. This data can be found here: https://www.ukbiobank.ac.uk.
