# Supplementary material for: Plasma proteomics and carotid intima-media thickness in the UK biobank cohort
Source: Front Cardiovasc Med. 2024 Oct 2;11:1478600. doi: 10.3389/fcvm.2024.1478600 (PMC11480011; doi:10.3389/fcvm.2024.1478600)
Supplement: Supplementary file 1 [file Datasheet1.docx]

**Supplementary File**

**Detailed Method**

### *Measurement of covariates*

For physical measures, we obtained systolic blood pressure (SBP), diastolic blood pressure (DBP), and anthropometric measures, including waist circumference, body mass index (BMI), calculated whole body fat and fat-free mass index (kg/m^2^), trunk fat and fat-free mass index. We included blood biochemistry variables, including total cholesterol, LDL cholesterol (direct), HDL cholesterol, triglycerides, apolipoprotein A (ApoA), apolipoprotein B (ApoB), lipoprotein A [Lp(a)], C-reactive protein, and glycated hemoglobin (HbA1c). Self-reported medical conditions were utilized, including diabetes and cardiovascular diseases which covered myocardial infarction, angina, stroke, and hypertension. Genetic information was leveraged directly from the UK Biobank, including polygenic risk scores (PRS) for cardiovascular disease (CVD), hypertension, ischemic stroke (ISS), and type 2 diabetes (T2D), as well as genetic principal components (PC). Full list of variables included is depicted as below:

| **Variable** | **UKBB field number code** |
| --- | --- |
| Proteome normalized protein expression (NPX) value | 30900 |
| cIMT measurement at instance 2 | 22670 to 22681 |
| cIMT measurement quality control indicators | 22682 to 22685 |
| Sex | 31 |
| age when attending the assessments | 21003 |
| Assessment center location of visits | 54 |
| Self-reported ethnic background ^a^ | 21000 |
| Townsend deprivation index | 22189 |
| Alcohol frequency | 1558 |
| Physical activity groups categorized based on the summed MET (Metabolic Equivalent Task) minutes per week for all activity ^b^ | 22040 |
| Waist circumference | 48 |
| Body mass index | 23104 |
| Whole body fat mass index ^c^ | 23100, 50 |
| Whole body fat free mass index ^c^ | 23101, 50 |
| Trunk fat mass index ^c^ | 23128, 50 |
| Trunk fat-free mass index ^c^ | 23129 |
| SBP | 4080 |
| DBP | 4079 |
| Apolipoprotein A | 30630 |
| Apolipoprotein B | 30640 |
| C-reactive protein | 30710 |
| Cholesterol | 30690 |
| Glycated haemoglobin (HbA1c) | 30750 |
| LDL cholesterol (direct) | 30780 |
| HDL cholesterol | 30760 |
| Lipoprotein A | 30790 |
| Triglycerides | 30870 |
| Diabetes diagnosed by doctor | 2443 |
| Vascular/heart problems diagnosed by doctor | 6150 |
| Medication for cholesterol, blood pressure or diabetes prescription ^d^ | 6177 |
| Statin use ^e^ | 20003 |
| Aspirin use | 6154 |
| Standard polygenic risk scores (PRS) for cardiovascular disease (CVD) | 26223 |
| Standard PRS for hypertension | 26244 |
| Standard PRS for ischemic stroke (ISS) | 26248 |
| Standard PRS for type 2 diabetes (T2D) | 26285 |
| Genetic principal components (PC) | 22009 |
| Follow up time ^f^ | 53 |

Footnote:

a: Ethnic background information was obtained from the self-reported touchscreen questionnaire. Those who identified as Asian, Asian British, Chinese, Indian, Pakistani, Bangladeshi, or any other Asian background were classified as Asian; Caribbean, African, or any other black background were classified as Black; Individuals who identified as British, Irish, or any other White background were classified as White; Finally, those who reported as White and Black Caribbean, White and Black African, White and Asian, or any other mixed background were classified as mixed; b: We defined high physical activity group as the summed Metabolic Equivalent Task (MET) score > 3000 minutes per week, MET score between 3000 to 600 minutes per week as moderate, < 600 minutes per week as low physical activity group; c. whole body fat mass index was calculated using whole body fat mass (kg) divided by the square of standing height (transformed to meter). The same method was applied to calculate whole body fat free mass index using whole body fat-free mass, trunk fat mass index using trunk fat mass, and trunk fat-free mass index with trunk fat-free mass; d. self-reported medication data was obtained on prescription medication use for the management of dyslipidemia, hypertension, or diabetes; e. We additionally identified statin use from the treatment/medication record and aspirin use from the field of medication for pain relief, which includes simvastatin, fluvastatin, pravastatin, velastatin, atorvastatin and rosuvastatin. f. Follow up time was calculated as the days between the date of attending initial assessment center and the date of image visit. A full cohort characteristic is described in **Supplementary Table 1.**

### *Measurement of Outcome*

We included individuals who had full cIMT measurements at instance 2 (48,597 participants) and who did not fail any sample quality control indicators. CIMT measurements available from the UK Biobank Imaging Enhancement Study were measured using a CardioHealth Station ultrasound system. Participants were placed in supine position, with their head rotated at 45°. CIMT of the right common carotid artery was imaged using automated edge‐detection software at the angle of 150° and 120°, and the left common carotid artery at 210° and 240°. Sonographers assessed CIMT scan quality against predefined UK Biobank standards. Regular internal review of all scans by senior radiographers and external validation of randomly selected scans at independent facility was conducted to ensure the quality. Individuals with cIMT measurements that did not pass the quality control, either with any cIMT values of zero or as indicated by the corresponding quality control flags were excluded from this study.

### *Linear Regression Model for Non-proteomic factors*

We applied unadjusted (univariate) and adjusted linear regression models to screen for non-proteome risk factors for cIMT. (**Supplementary table 3**) We selected five basic non-modifiable characteristics, including sex, age at baseline, age at imaging visit, center of imaging visit, and ethnicity groups incorporated as core confounders to be adjusted for. A total of 37 variables had unadjusted p values, which passed univariate screening at crude P< 0.05, and 25 variables, which passed adjusted linear regression screening at crude P< 0.05.

### *Linear Regression Model for Proteins*

To evaluate the association between each plasma protein and the outcome of cIMT under different levels of confounding adjustment, we applied multiple adjusted linear regression models (LM) which ran independently on each proteome and were adjusted for all 26 variables identified from the previous stepwise AIC screening process: adjusted for five basic non-modifiable characteristics (LM 1); further adjusted for genetic components including polygenic risk score (PRS) for CVD, PRS for HTN, and genetic PC5 (LM 2); further adjusted for health behaviors including binary ever smoked status and physical activity categorized by MET (LM 3); further adjusted for physical measures including SBP (mm Hg), DBP (mm Hg), waist circumference (cm), body fat mass index (kg/m^2^), trunk fat mass index (kg/m^2^), and trunk fat free mass index (kg/m^2^) (LM 4); and finally, additionally adjusted for blood biomarkers and medical conditions including apolipoprotein A (g/L), apolipoprotein B (g/L), and cholesterol lowering medication use (LM 5).

### *LASSO Regression*

The cIMT was predicted as a continuous outcome. A full rank parameterization of the predictor data was implemented, and dummy variables for all levels of categorical factors were created, excluding the reference category. We built the LASSO model using 10-fold cross-validation to find the optimal lambda, which minimizes test mean squared error (MSE). To benchmark the predictive ability of the five linear regression models we built before, we also evaluated their predictive ability by calculating the R^2^ and RMSE of models within training and testing sets.

### *Gene Set Enrichment Analysis*

The full 3795 canonical pathways (CP) from the curated gene sets (C2 collection, version 2023.2) as well as the full 50 pathways from the Hallmark gene sets (version 2023.2) in the Molecular Signatures Database (MSigDB) were investigated in this study. CP collection encompasses gene sets from various domain resources, including BioCarta, KEGG, Reactome, PID, and Wikipathways. With the default parameters of GSEA, after filtering from the gene sets, any gene not in the expression dataset along with curated gene sets with a size smaller than 15 or larger than 500 were excluded from the analysis. We calculated the signed log-transformed p-values from the linear regression model adjusted for minimal non-modifiable characteristics for each protein and cIMT associations, then ranked them as input for GSEA software (v4.3.2). Enrichment score, which represents the degree to which a gene set is overrepresented at the top or bottom of the ranked genes, was then calculated as the maximum deviation from zero encountered during each round of the running-sum statistic from the ranked list of genes.

### **Supplementary Result**

### *Gene Set Enrichment Analysis*

After applying gene set size filter, 327 gene sets were used in GSEA canonical pathway analysis. We found 290 gene sets positively linked to cIMT, 9 of them reached FDR < 0.25. Some of the top positively overrepresented gene sets were enriched for core signaling pathways involving T-cell receptor signaling, infection response, epidermal growth factor receptor (EGFR) signaling, angiopoietin receptor signaling, VEGFA-VEGFR2 signaling, membrane trafficking, and the cell cycle. In contrast, 37 gene sets were found to be downregulated in cIMT, which were enriched for pathways involving keratinization, the hair follicle and neuronal systems, metabolism of glycosaminoglycan, heparan, fatty acid, regulation of Insulin-like Growth Factor (IGF) transport and uptake, and peroxisome proliferator-activated receptors (PPAR) signaling.

Similarly, 36 out of 50 gene sets were used in hallmark gene set analysis. Three hallmark gene sets showed significant at FDR-q < 0.25 level, including pathways of myogenesis, allograft rejection, mitotic spindle assembly. (**Supplementary Table 10 and Supplementary Figure 3 and Figure 4**)

### **Supplementary Discussion**

### *Extended discussion on GSEA results*

Our GSEA applied to circulating plasma proteins provides some hints to the molecular processes involved in cIMT. Using the Molecular Signatures Database canonical pathways collection, we identified 290 upregulated and overrepresented canonical pathways including 9 gene sets significant at FDR < 0.25, mostly involving core signaling in hemostasis (i.e., monoglyceride lipase), angiogenesis (i.e., insulin-like growth factor-binding protein 7), and vesicle-mediated transport (i.e., vesicle trafficking).^1-4^ Monoglyceride lipase, which also participates in the regulation of fatty acids, has been reported to be associated with an increased risk of cIMT in a gene-smoking interaction GWAS study in sub-Saharan Africans.^3^ Vesicle trafficking 1 is a plasma protein, which also carries an increased risk for cIMT in the gene-smoking interaction GWAS study.^5^ Notably, VTA1 is involved in the budding of HIV virion, which may explain the enriched HIV pathway in our GSEA analysis^6,7^ A lesser number of canonical pathways were downregulated in cIMT, however, versican core protein (VCAN) was found to be core enrichment that contributed to most of these pathway findings, including glycosaminoglycan metabolisms and ECM proteoglycans. These findings are consistent with a recently published GWAS from UKBB, which identified that alleles mapped to VCAN were associated with decrease in mean cIMT and observed an enrichment of gene sets in extracellular organization and the platelet-derived growth factor signaling pathways.^1,8^ Together, these studies again pointed out the important role of ECM components in atherosclerosis, but further studies are needed to allow for more robust conclusions in this respect given our limited power in these analyses.

### *References:*

1. Yeung MW, Wang S, van de Vegte YJ, et al. Twenty-Five Novel Loci for Carotid Intima-Media Thickness: A Genome-Wide Association Study in >45 000 Individuals and Meta-Analysis of >100 000 Individuals. *Arterioscler Thromb Vasc Biol*. Apr 2022;42(4):484-501. doi:10.1161/atvbaha.121.317007

2. Boua PR, Brandenburg JT, Choudhury A, et al. Genetic associations with carotid intima-media thickness link to atherosclerosis with sex-specific effects in sub-Saharan Africans. *Nat Commun*. Feb 14 2022;13(1):855. doi:10.1038/s41467-022-28276-x

3. Boua PR, Brandenburg JT, Choudhury A, et al. Novel and Known Gene-Smoking Interactions With cIMT Identified as Potential Drivers for Atherosclerosis Risk in West-African Populations of the AWI-Gen Study. *Front Genet*. 2019;10:1354. doi:10.3389/fgene.2019.01354

4. Schaefer CF, Anthony K, Krupa S, et al. PID: the Pathway Interaction Database. *Nucleic Acids Res*. Jan 2009;37(Database issue):D674-9. doi:10.1093/nar/gkn653

5. Ward DM, Vaughn MB, Shiflett SL, et al. The role of LIP5 and CHMP5 in multivesicular body formation and HIV-1 budding in mammalian cells. *J Biol Chem*. Mar 18 2005;280(11):10548-55. doi:10.1074/jbc.M413734200

6. Grunfeld C, Delaney JA, Wanke C, et al. Preclinical atherosclerosis due to HIV infection: carotid intima-medial thickness measurements from the FRAM study. *Aids*. Sep 10 2009;23(14):1841-9. doi:10.1097/QAD.0b013e32832d3b85

7. Shrestha S, Irvin MR, Taylor KD, et al. A genome-wide association study of carotid atherosclerosis in HIV-infected men. *Aids*. Feb 20 2010;24(4):583-92. doi:10.1097/QAD.0b013e3283353c9e

8. Strawbridge RJ, Ward J, Bailey MES, et al. Carotid Intima-Media Thickness: Novel Loci, Sex-Specific Effects, and Genetic Correlations With Obesity and Glucometabolic Traits in UK Biobank. *Arterioscler Thromb Vasc Biol*. Feb 2020;40(2):446-461. doi:10.1161/atvbaha.119.313226

**Supplementary Figure 1. Variance explained (R^2^) of the Linear regression models ^a^ performed in the testing datasets.**


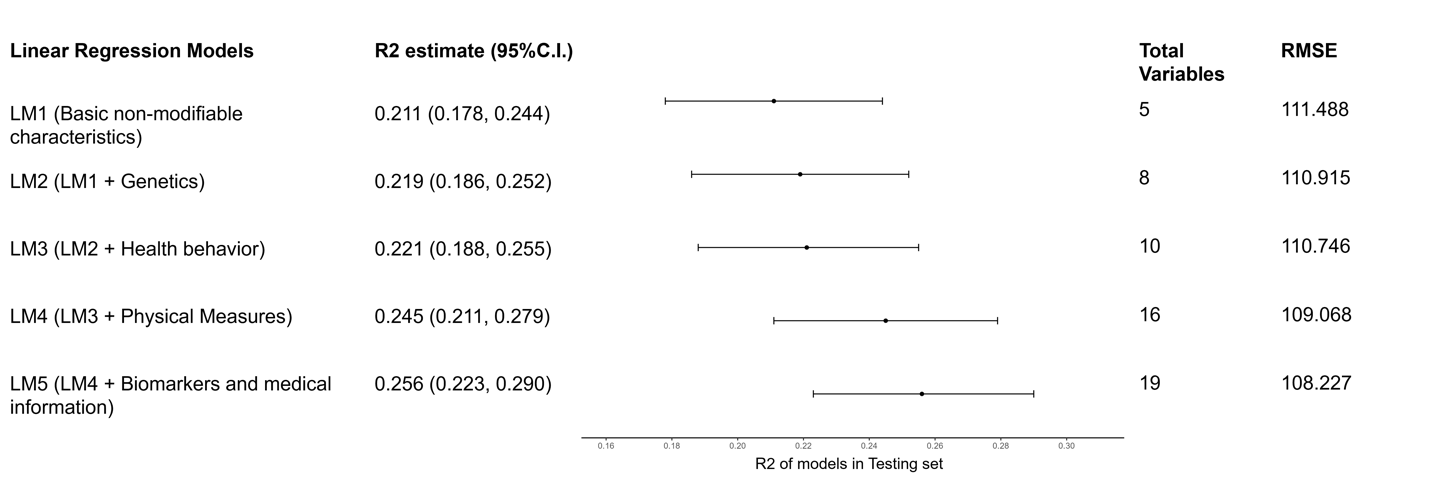


Footnote:

a. Linear regression Model 1 (LM1) adjusted for sex, age at baseline, age of cIMT measurement, image visit center, and ethnicity. Model 2 (LM2) adjusted for variables in LM1 plus PRS for CVD, PRS for HTN, and genetic PC5. Model 3 (LM3) further adjusted for binary ever smoked and physical activity categories. Model 4 (LM4) further adjusted for SBP (mmHg), DBP (mmHg), waist circumference (cm), body fat mass index (kg/m^2^), trunk fat mass index (kg/m^2^), and trunk fat free mass index (kg/m^2^). Finally, Model 5 (LM5) further adjusted for ApoA (g/L), ApoB(g/L) and cholesterol lowering medication use.

Abbreviations: RMSE: root mean square error.

**Supplementary Figure 2. Cross-validation curves with upper and lower standard deviation curves along the 𝜆 sequence of LASSO models.**

a. Models performed without selected covariates forced in the models.


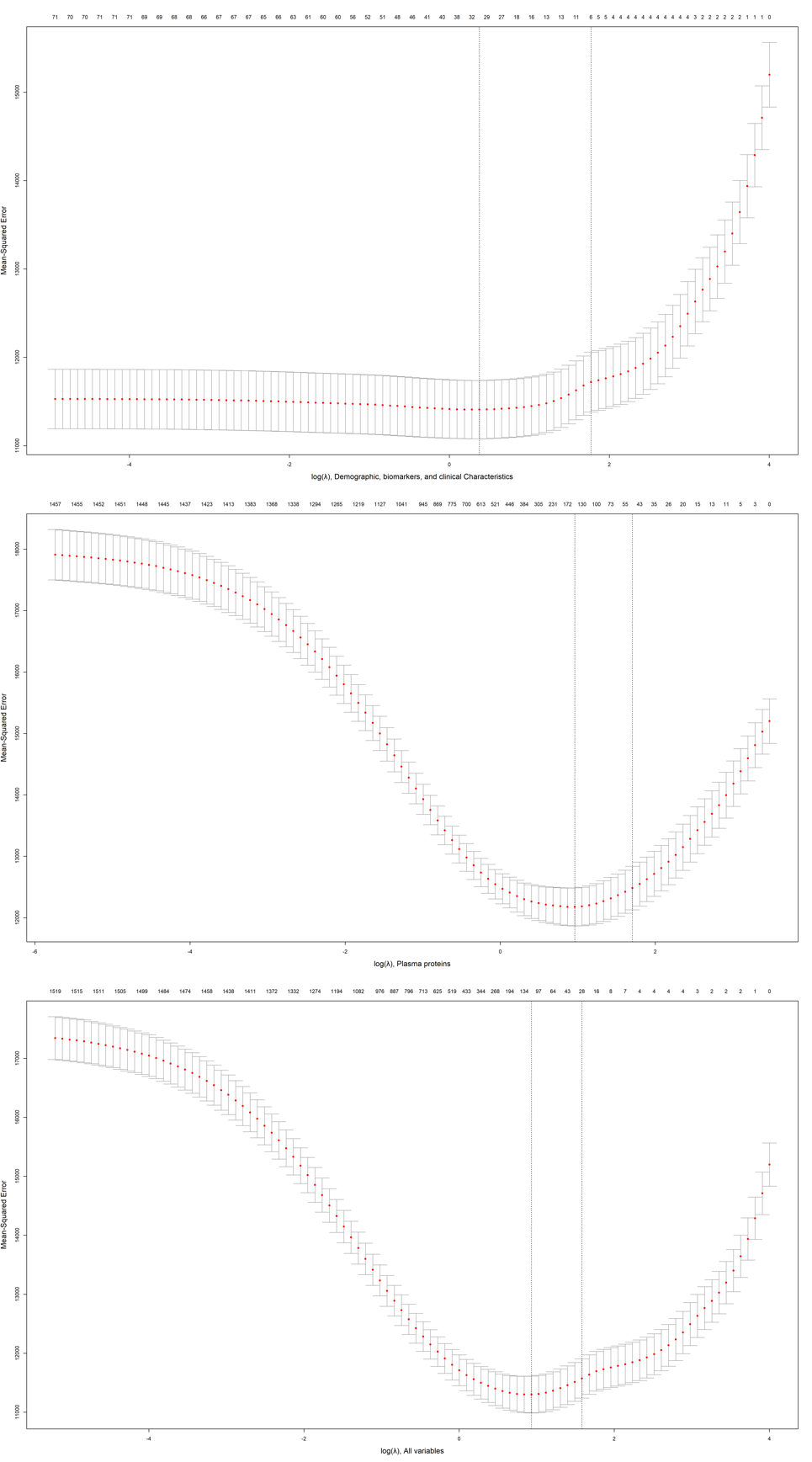


b. Models performed with selected covariates forced in the models.


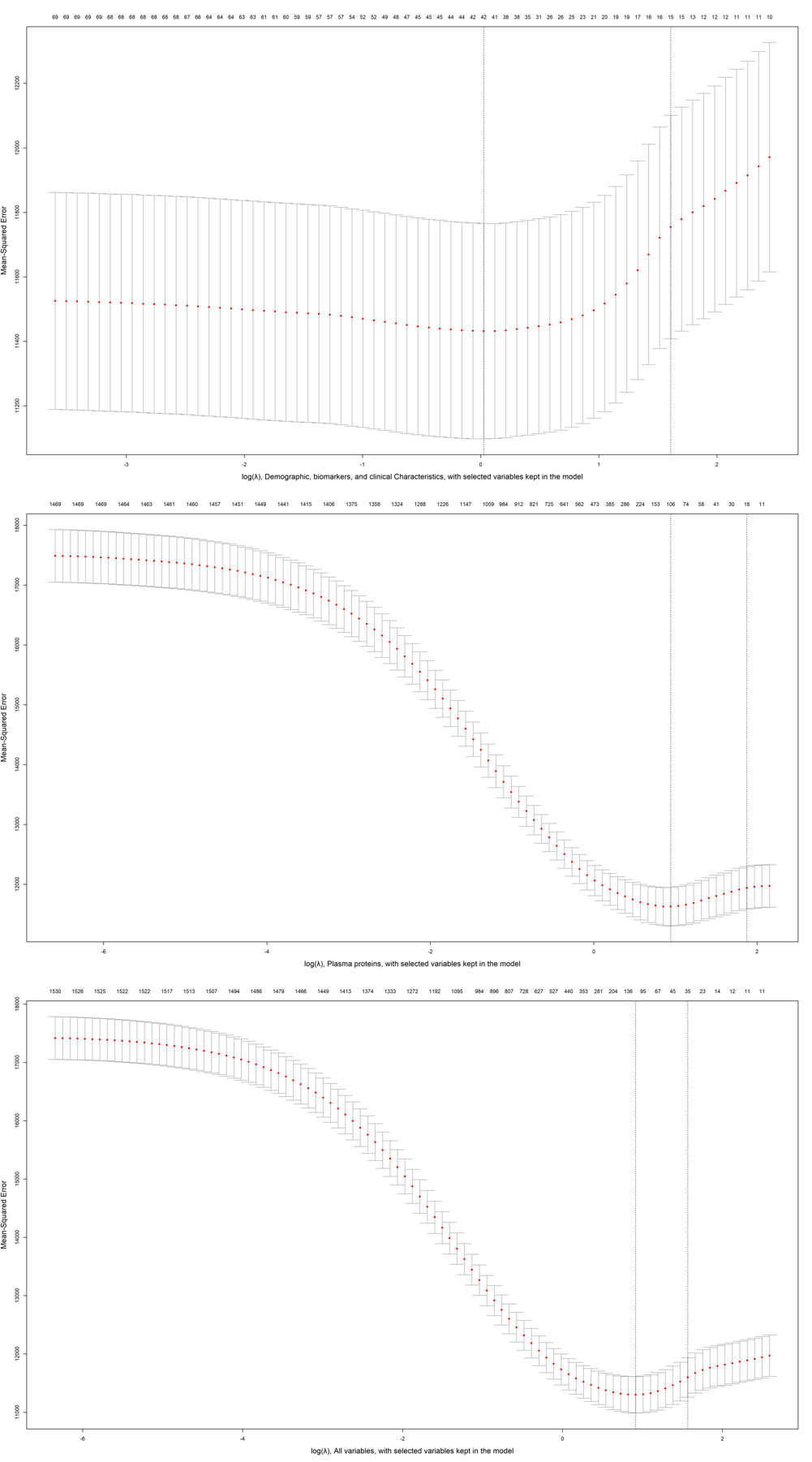


**Supplementary Figure 3. Top overrepresented canonical pathways in GSEA for protein**


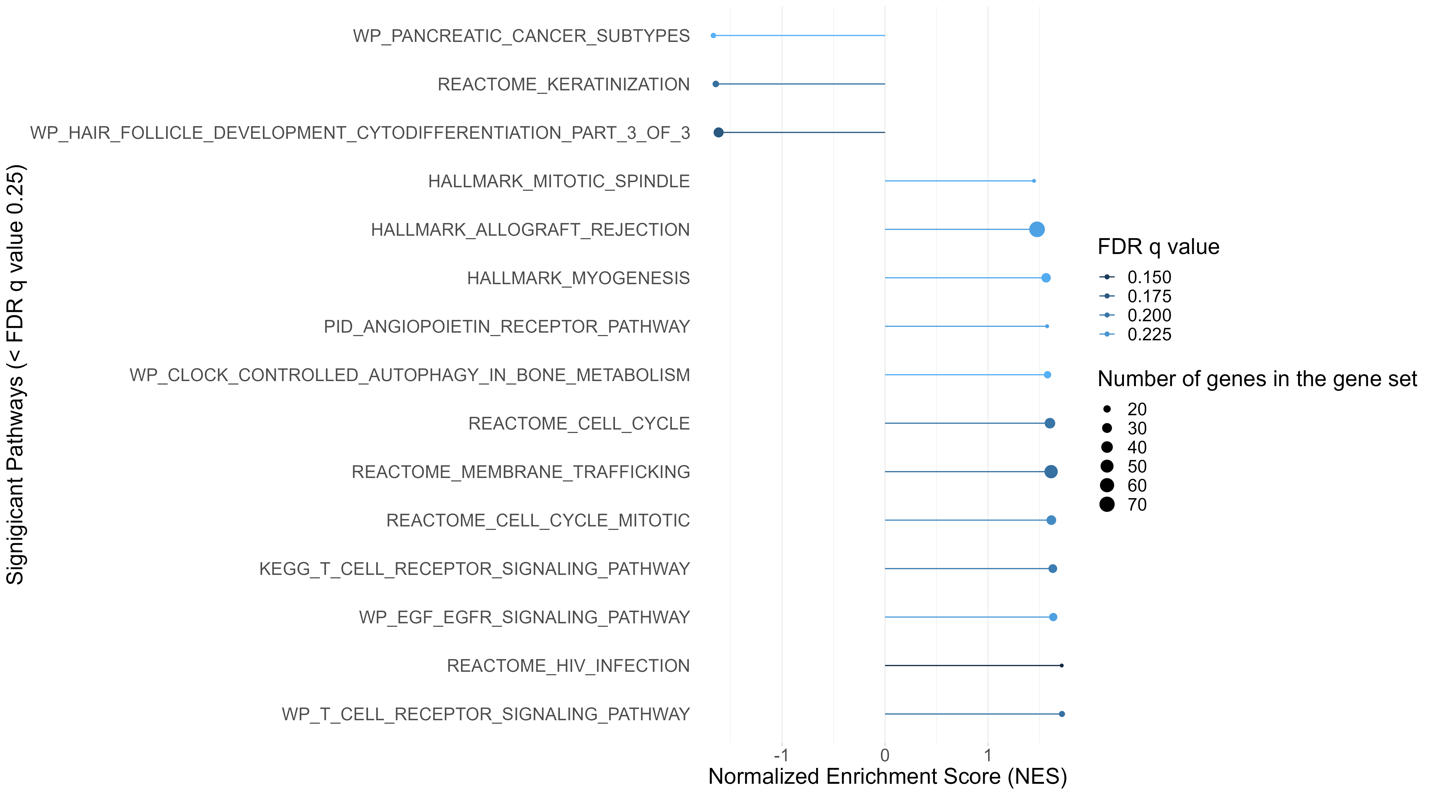


Footnote: Selected canonical biological pathways under FDR q value of 0.25. Full GSEA results are shown in supplementary table 10.

Abbreviations: GSEA: gene set enrichment analysis; FDR: False discovery rate; NES: Normalized enrichment score.

**Supplementary Figure 4. GSEA statistics and enrichment results.**


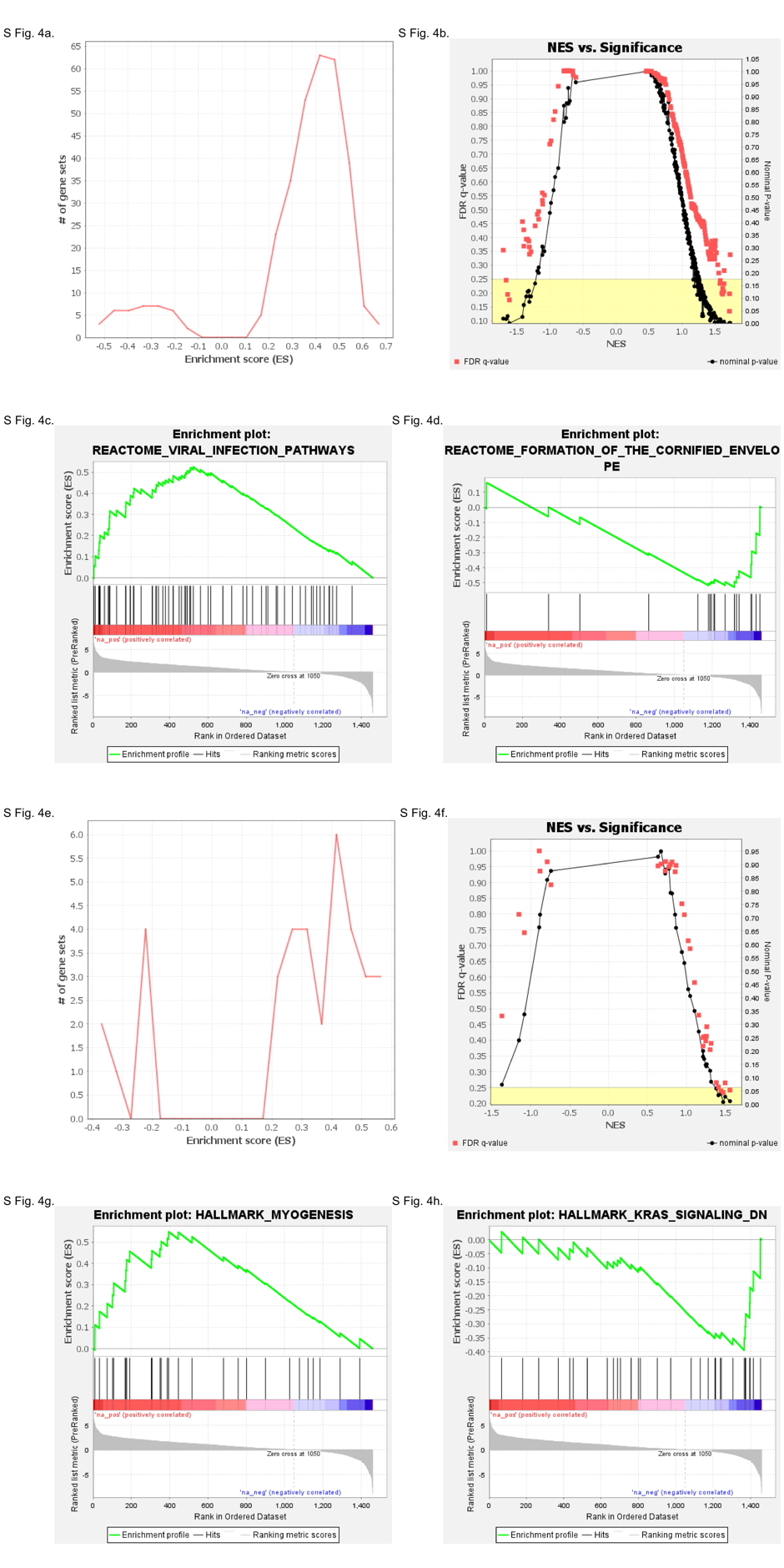


Footnote:

4a. The histogram of enrichment scores across canonical pathway gene sets suggesting that most of the enriched gene sets were positively associated with cIMT. 4b. Plot of nominal p values and FDR q-values across NES among canonical pathways, depicting the number of enriched gene sets that are significant. 4c. The canonical pathway with the highest absolute positive NES. (Reactome viral infection pathways, NES = 1.73, FDR q-value = 0.338) 4d. The canonical pathway with the highest absolute negative NES. (Reactome formation of the cornified envelope pathways, NES = -1.71, FDR q-value = 0.354) 4e. The histogram of enrichment scores across hallmark gene sets. 4f. Plot of nominal p values and FDR q-values across NES among hallmark gene sets. 4g. The hallmark gene set with the highest absolute positive NES. (Myogenesis, NES = 1.56, FDR q-value = 0.242) 4h. The hallmark gene set with the highest absolute negative NES. (KRAS signaling downregulation, NES = -1.37, FDR q-value = 0.477)

Abbreviations: GSEA: gene set enrichment analysis; FDR: False discovery rate; NES: Normalized enrichment score.
